# Comparison of resistance training vs static stretching on flexibility and maximal strength in healthy physically active adults, a randomized controlled trial

**DOI:** 10.1186/s13102-024-00934-1

**Published:** 2024-06-28

**Authors:** Morten Rosenfeldt, Nicolay Stien, David G. Behm, Atle Hole Saeterbakken, Vidar Andersen

**Affiliations:** 1 Faculty of Education, Arts and Sports, Western Norway University of Applied Sciences, PB 133, Sogndal, 6851 Norway; 2 School of Human Kinetics and Recreation, Memorial University of Newfoundland, St. John’s, Newfoundland and Labrador, Canada

**Keywords:** Sit and reach, Range of motion, Strength training, Deadlifts, Lower back, Isometric

## Abstract

**Background:**

The aim of the present study was to compare the effects of resistance training through full range of motion and static stretching (SS) of the hip and lower back extensors on flexibility and strength in healthy, physically active, adults.

**Methods:**

Eighteen participants (age: 24.2 ± 3.0 years, body mass: 71.3 ± 8.9 kg, height: 172.8 ± 7.5 cm) were randomly assigned to either a Resistance Training (RT) (*n* = 6), SS (*n* = 6), or control (CON) group (*n* = 6). The sit & reach (S&R) flexibility test and maximum isometric straight legged deadlift (ISLDL) at 95% and 50% range of motion (ROM) were tested pre- and post-intervention with significance set at *p* < 0.05. Both groups conducted four to eight sets per session. Within each set, the RT group performed eight repetitions each lasting four seconds, while the SS group stretched continuously for 32 s. The rest periods between each set were 60–90 s. Consequently training volume and rest times were matched between the groups.

**Results:**

The RT and SS groups achieved significant, large magnitude improvements in the S&R test compared to the CON group (*p* < 0.01 g = 2.53 and *p* = 0.01, g = 2.44), but no differences were observed between the RT and SS groups (*p* = 1.00). Furthermore, the RT group demonstrated a larger improvement in 50% and 95% ROM ISLDL compared to SS (*p* < 0.01, g = 2.69–3.36) and CON (*p* < 0.01, g = 2.44–2.57).

**Conclusion:**

Resistance training through a full ROM was equally effective as SS for improving S&R flexibility, but improved hip- and lower back extensor strength more than SS and the CON. The authors recommend using large ROM resistance training to improve hip and lower back extensor flexibility and muscle strength.

**Trial registration:**

ISRCTN88839251, registered 24. April 2024, Retrospectively registered.

## Background

Flexibility is the movement of a joint and the lengthening of the surrounding muscles [1]. The range of motion (ROM) is a measure of the flexibility and used as significant measurement clinically to reduce pain, manage daily living activity, and to reduce the risk of musculotendinous injuries (primarily with explosive change of direction actions) [2–5]. Typically, stretching is used to achieve increasing flexibility and ROM [1, 6, 7]. From a biomechanical perspective, stretching can be categorized into two mechanical sources: passive and active tension [8]. Passive tension occurs when the connective tissue is lengthened beyond its resting length and active tension occurs from the contractile effects from the interaction of actin and myosin filaments [8].

Stretching, either static or dynamic, has proven effective to increase flexibility and ROM although methods of measuring still vary between studies [6, 8–11]. Importantly, there is a debate how much stretching’s positive change on flexibility and ROM is an adaptation in form of structural change in muscle length, fascia length and connective tissue. Additionally, there is discussion to what extent this impact is attributed to adaptations in stretch tolerance or passive stiffness [8, 10, 12, 13]. Furthermore, stretching has been an important part of exercise warm-up and cooldown routines [14]. Prolonged static stretching (SS) has been met with criticism when performed prior to the main activity or exercise due to the potential performance impairments, but recent findings suggest that less than 60 s of SS per muscle group do not cause performance decrements [8, 14–16]. Instead, a recent meta-analysis showed that chronic SS has the potential for small positive improvements on muscle strength [17]. It is important to note that multiple studies included in the meta-analysis were done with elderly and/or sedentary individuals, which makes it uncertain if similar adaptations would happen in young and/or trained individuals.

Stretching might not be the only way to increase flexibility and ROM. While resistance training (RT) is generally associated with increased muscle cross-sectional area, force generation and neural adaptations, RT has demonstrated increased flexibility in a variety of joints and muscles [18–24]. Due to the small pool of evidence, the heterogeneity of study designs, and the populations included in trials on RT’s effect on flexibility, the evidence from individual original studies is contradictory and invites further research [6, 18]. For example, Aquino et al. [25] demonstrated that eight weeks of RT three times a week in a lengthened muscle position had no effect on flexibility among participants with limited ROM in the hamstrings. In contrast, Simäo et al. [19] reported increased flexibility after 48 RT workouts spread over 16 weeks [19]. However, the participants were sedentary elderly women. Further, Alegre et al. [26] showed that dynamic RT increased vastus lateralis fascicle length, muscle thickness, and squat strength among male physical education students. Accordingly, RT combined with stretching has demonstrated increased stiffness in tendon structures and the viscosity of the tendon structures without affecting the elasticity [27]. Although some individual studies have reported contrasting results, a systematic review and meta-analysis of the literature by Alizadeh et al. [28] revealed similar ROM increases for both RT and stretching for both sexes with overall moderate magnitude increases.

There exist multiple reason why time-efficient and effective RT as well as flexibility training programs is called up-on. The perceived level of time and effort is a significant barrier in preventing people performing regular RT or flexibility training [29]. A recent meta-analysis found seven studies that directly compared RT and SS and concluded that both interventions improved range of motion to a similar extent [28]. However, none of these matched training volume or stretch intensity between the different interventions, which are fundamental variables for training effects [30, 31]. Furthermore, four of the studies did not measure strength [32–35], which obfuscate the effect of the resistance and stretching interventions on muscular strength. Therefore, the present study aimed to investigate the effects of eight weeks of RT or SS, of the hip- and lower back extensor, on flexibility- and maximal strength when training time and stretch intensity were matched between groups in healthy, physically active, young adults. Both training groups (i.e., RT and SS) were compared to a control (CON) group. It was hypothesized that the RT and SS group would increase flexibility similarly and more than CON. Further, RT was hypothesized to increase maximal strength more than both SS and CON group.

## Methods

### Experimental approach to the problem

This study used a randomized control trial design to determine potential differences in flexibility and maximal strength changes between the groups. The testing was conducted in a controlled environment (i.e., Strength Laboratory) at the university. The same investigator conducted all testing and supervised training sessions. Participants were told to refrain from strenuous exercise involving the lower extremities 48 h prior to testing and during the intervention period. Participants performed three test rounds during the trials. The first visit to the laboratory was to familiarize the participants with the test procedures. The second visit was the pre-test, where all tests were recorded. At least 48 h of rest was provided between familiarization and pre-test visits. The third visit was the post-test performed after 8 weeks of intervention, and 48–96 h after the last training session of the intervention. The testing was conducted in the strength-lab of Western Norway University of Applied Sciences, Campus Sogndal.

### Subjects

Based on the improvement in the S&R flexibility test in Simäo et al. [19] and with a α = 0.05 and β = 0.80, the sample size of six participants in each group appeared to be necessary to detect significant difference in the S&R flexibility test between the intervention and control groups. The recruitment started 30/06/2022 and ended 15/08/2022. Participants were recruited at public spaces of the university campus, through information meetings, Facebook groups and known associates in the author’s personal network. To be included participants had to have an active lifestyle according to Physical Activity Guidelines for Americans [36] before the start of the trial. Further, the participants should have little to no previous experience with deadlifting, and have no illness or injury to prevent them to conduct the training and testing. Eighteen men [5] and women [13] between the ages of 19 and 30 years were recruited and participated in this study during the autumn of 2022. After the pre-test protocol was completed, the participants were randomized into three groups: Resistance training (RT, *N* = 6), Static stretching (SS, *N* = 6) and Control (CON, *N* = 6) by drawing notes from a hat. The allocation process was generated by a third person. For a description of the different groups see Table 1. Participants were informed in writing and verbally of test and intervention protocols as well as potential risks and benefits and signed an informed consent form. The participants gave their consent to the use of anonymous data for publication of the study results. According to national legislation, an ethical approval from the regional ethical committee was deemed unnecessary (“Act on ethics and integrity in research” and “Act on medical and health research” (www.lovdata.no)). Importantly, the study was conducted ethically according to the standards described by the latest Helsinki Declaration and the universities ethical guidelines. Further, procedures for gathering and saving personal data was evaluated and approved by National Centre for Research Data (reference nr: 750,979) before the trial start. The study was registered retrospectively as a clinical trial (ISRCTN88839251, 24/04/2024). The study adhered to the CONSORT guidelines [37].

**Table 1:** Antropometric data of the groups (mean ± standard deviation)

	Control (*N* = 6)	Static stretching (*N* = 6)	Resistance training (*N* = 6)
Sex (F/M)	5/1	5/1	3/3
Age (years)	24.8 ± 2.6	24.1 ± 4.0	23.7 ± 2.9
Height (cm)	177 ± 10	171 ± 4	171 ± 7
Body mass (kg)	72.9 ± 10.8	71.4 ± 9.7	69.7 ± 7.4

F = female, M = male, Cm = centimeters, Kg = kilograms

### Procedures

In the familiarization test, the participants` body mass and height was measured before the participant’s maximum standing hip flexion was measured. The participants flexed their hip as far as possible while keeping their back in a natural straight position and knees extended, but not locked. The maximal hip ROM was measured with a universal goniometer and used to calculate the 95% and 50% of full ROM. The participants then performed a short general warmup following recommendations for the ‘Isometric Mid-thigh Pull’ (IMTP) [38] and for the ‘Sit and Reach’ (S&R) flexibility test [39]. The warmup consisted of five minutes of self-selected light- to moderate intensity running (participants was told they should be able to talk brief, but not long sentences), 12 bodyweight squats, 12 reverse lunges and some light dynamic stretching consisting of 12 standing good mornings and 12 cross-body toe reaches.

#### The sit-and reach test

The S&R test was utilized to measure hip- and lower back extensor flexibility and has shown high reliability [11]. Before starting the procedures, the participants were instructed on how to perform the test. The participants sat on the floor with extended knees, with heels touching the S&R box with their feet approximately hip width apart [39]. Participants were instructed to reach slowly forward with extended arms with the palms facing down. The participants` hands were overlapping equally so one hand was not further forward than the other and the fingertips were in contact with the measuring part of the S&R box. The participant’s reached as far forward as possible and held the furthest possible position for two seconds. The participants repeated this process three times with one minute rest between attempts, and the best score was included in the analysis. The participants were instructed to let their head drop between their arms and exhale while reaching forward, without holding their breath. The score was measured as the maximal distance (cm) the participants could reach forward [39]. The scale on the S&R box started 23 cm (9 inches) proximal to the feet. Intraclass correlation coefficient (ICC) score for the S&R test between attempts in pre-test was excellent (0.99, CI: 0.97–0.99), and mean coefficient of variation (CV) was acceptable (7.93%) [40].

#### Isometric strength tests

The isometric straight legged deadlift test (ISLDL) was used to measure the hamstrings and lower back strength at 95% and 50% standing hip ROM. The ISLDL was performed 5 min after the S&R test. The testing procedures primarily followed standardizations and guidelines for the IMTP [38], with adjustments to fit the investigated ISLDL as no standardization for an identical test exists. The test was performed with extended knees, without overextending the knee joint, and with a natural straight back. Minimum movement and spinal flexion in lumbar region were allowed. The bar height was adjusted to fit 95% and 50% maximum standing hip ROM, and the bar height was recorded for further tests. The 95% of ROM was tested first for all participants and at all testing occasions. The bar height was adjustable in 2.2 cm increments, so bar height was never more than 1.1 cm from the intended hip flexion. To measure the maximal strength of the hamstrings and lower back, the participants stood on two independent Force Plates (Musclelab Force Plate Sensors, Ergotest Innovation A/S, Porsgrunn, Norway, sampling frequency of 1000 Hz) which were synchronized by a Musclelab Data Synchronization Unit (Ergotest Innovation A/S, Porsgrunn, Norway). The participant’s heel position was chosen at approximately hip width apart with the bar over midfoot, and toe position where participants felt most comfortable. The position was recorded for identical position in all tests. Participants were encouraged to use a mixed grip on the handle to make grip strength less of a factor in testing [33]. Before maximal effort, the participants did three warm-up attempts at 50%, 75%, and 90% of perceived maximum effort with 60 s of rest between each attempt, in accordance with recommendations for the IMTP [38].The participants were instructed into the correct position with verbal cues; ‘straighten back’, ‘straighten knees’ ‘weight on midfoot’, ‘chest up’, and ‘chin down’. For the maximum effort attempts, participants were given a countdown 3, 2, 1 PULL. The participants then did three attempts at 100% effort with 90 seconds rest between sets, of which their best result was used in the analysis. After testing at 95% hip flexion, the bar height was adjusted to match 50% hip flexion. Participants did one warm-up set at 75% effort at 50% hip flexion, before doing three pulls of 100% effort which their best attempt was used in the analysis. Participants were encouraged to keep pulling for four seconds of maximum effort of which the best three seconds (highest mean force output) were used in the analysis. After each attempt, the force cells were reset. The results were analysed using a commercial software (Ergotest Innovation A/S, Porsgrunn, Norway). Further, ICC score for both the 95% and 50% ROM ISLDL between attempts were excellent reliability (95%; ICC 0.99, CI: 0.96–0.99, CV = 4.80%), 50%; ICC 0.99, CI: 0.95–0.99, CV = 4.08%), respectively.

### Intervention

**Fig. 1:**
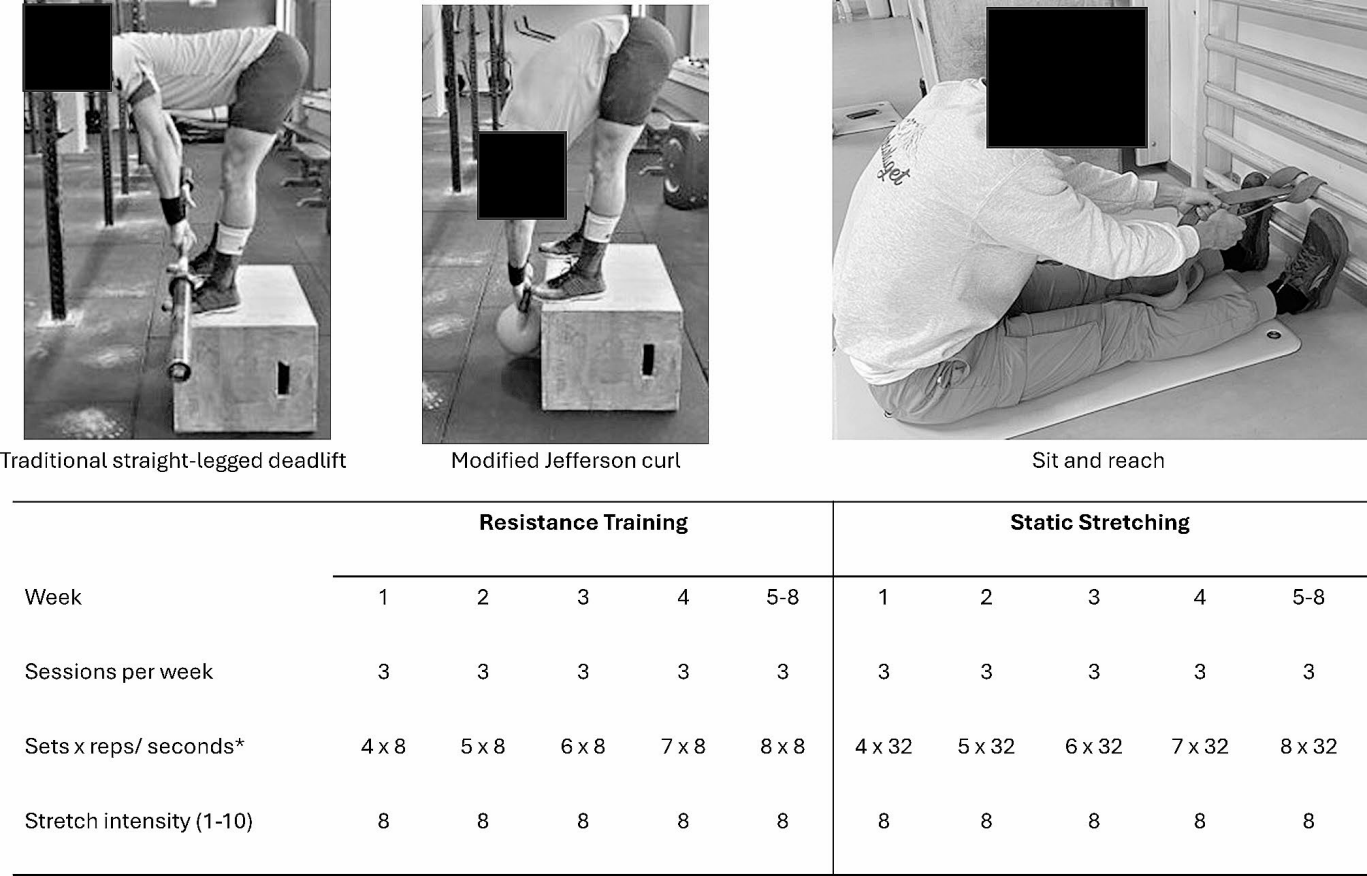
An overview of the training interventions. *Reps for resistance training and seconds for static stretching

The intervention lasted from the middle of August until the middle of December 2022. Both RT and SS groups performed their respective intervention for eight weeks with three sessions per week (Fig. 1). All groups were told to continue their normal activity level in addition to assigned training from their respective group. The same investigator was coaching the participants in RT and SS groups during the first two weeks of intervention. Furthermore, all participants had one extra coached session after 4 weeks of the intervention to monitor the participant’s progression, technique, and intensity level, i.e., stretch level and rating of perceived exertion (RPE). For the remaining weeks the participants shared their training log with the instructor in a digital document allowing the instructor to monitor the progression. The participants also had the instructor`s contact information and were encouraged to make contact if they had any questions regarding the training. The warmup for both SS and RT groups were 5 min of moderate-intensity biking, i.e., participants should only be able to conduct brief sentences. An important issue when trying to compare SS’ and RT’s effect on flexibility, is how to match the SS- and RT stretch intensity between groups, which to the authors knowledge have not been accomplished previously. Therefore, we tried to match the stretch intensity between SS and RT groups in discomfort felt from stretching [9, 10, 41]. Discomfort in the present study was used as a subjective perception of stretch intensity and maximal torque tolerance, which made it possible to subjectively control and match the intensity between the two training groups. Further, total time and sets stretched in SS were matched time, sets and repetitions from RT group, by supervising each repetition to be four seconds (3 s eccentric and one second concentric contraction, see Fig. 1).

#### Resistance training protocol

**Fig. 2:**
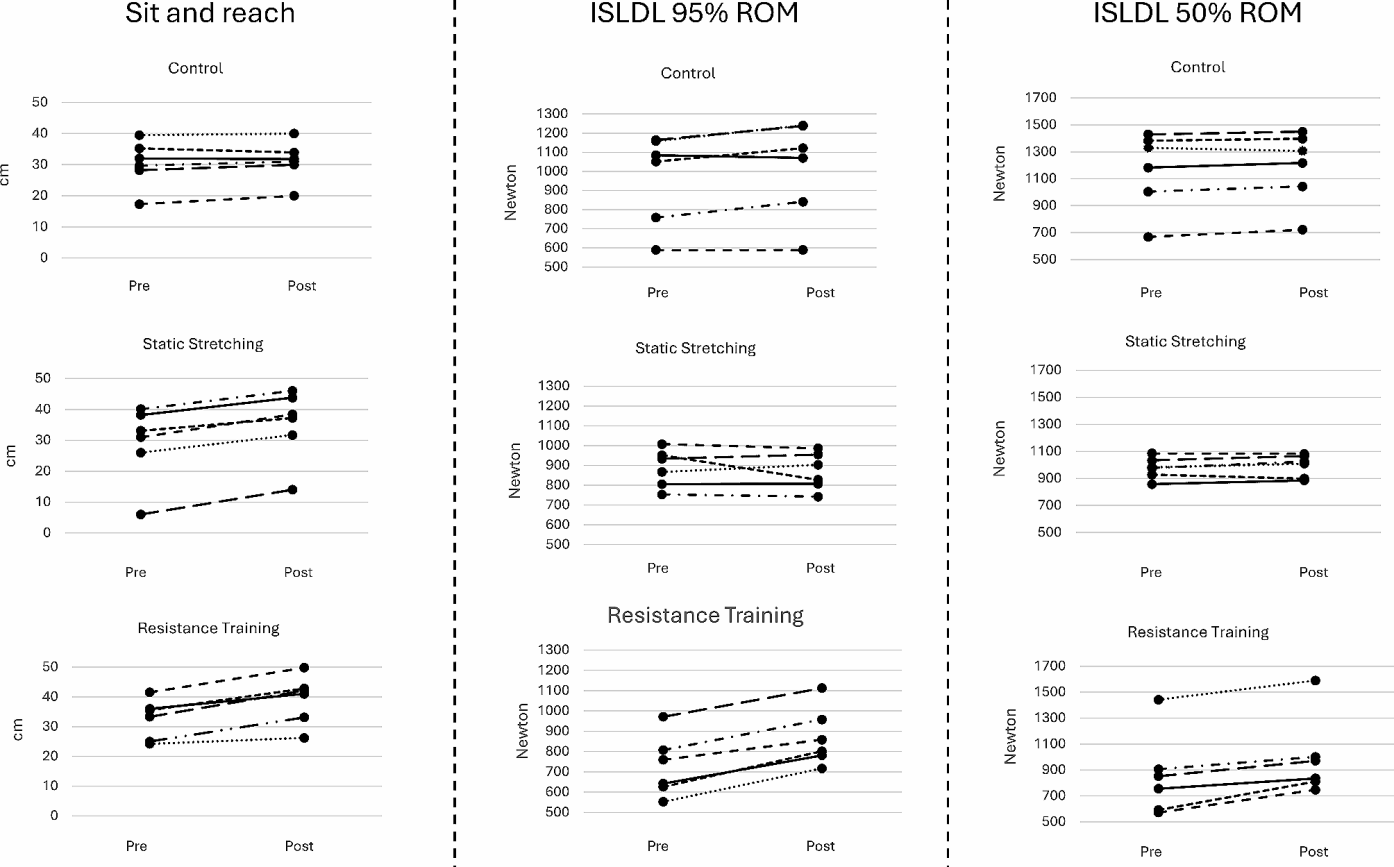
Individual data, from the Resistance Training-, Static Stretching- and the Control group, in the flexibility and strength tests. Each line represents one individual. ISLDL = isometric straight legged deadlift, ROM = range of motion

Participants performed two straight-legged deadlift exercise variants (traditional straight-leg deadlift and a modified Jefferson Curl). Both exercises were performed standing elevated on a box to give the possibility of increasing ROM in the lift (Fig. 2). The modified Jefferson Curl was performed with a kettlebell. Participants lowered the kettlebell while flexing at the hip keeping their vertebral column straight and knees nearly fully extended in the first half of the movement and then flexion of the vertebral column in the second half (Fig. 1). Flexion in the spine was encouraged to increase ROM. The second exercise was a traditional straight-legged deadlift performed with a barbell (Fig. 1). The participants were told to keep their back straight and knees nearly extended throughout the whole movement. In both exercises, the participants lowered the weight in an eccentric contraction for three seconds then came back up in a concentric contraction for one second, for a total of 4 s per repetition, 32 s per set to match the work time with SS-group. This tempo could be defined as moderate and has been recommended for novice and intermediate practitioners [30]. It also allowed the participants to control the stretch intensity during the eccentric contraction. Rest periods were 60–90 s between sets. Participants were told to reach a level of discomfort/intensity from stretching the hamstrings of eight on a visual analogue scale (VAS) from 0 to 10 to [9, 10]. Here, an intensity of 0 indicated no discomfort, and discomfort of 10 indicated the worst discomfort imaginable. This was done to match the intensity to the SS-group. In terms of loading, for the first four weeks of intervention, participants in RT group were told to reach an RPE of 8 (1–10 scale were 1 is no exertion and 10 is maximum exertion) after each set in both exercises. If an RPE of 8 was not reached, the weight was increased. This was done to focus the participants on performing correctly and getting accustomed to the movements and not focus on increasing the weight of the kettlebell and barbell unnecessarily. The last four weeks, the goal was to reach a minimum of 75% of the 95% ROM isometric straight-legged deadlift pre-test for the straight-legged deadlift. For the modified Jefferson Curl, the goal continued to be an RPE of 8 after each set. In both exercises, participants were coached verbally and visually to maximize hamstrings lengthening by focusing on hip hinging and getting the abdomen towards the upper thigh. All participants were novice deadlifters. Therefore, programming for the RT intervention protocol started with reduced weights to be precautionary and protect participants from injury. As the technique developed, the load was increased to match the prescribed RPE. Importantly, in the first sessions the instructor emphasized helping the participants to understand and rate their exertion. Intended exertion was typically achieved within the first two to three sessions. The trial utilized a progressive approach. Additionally, participants were explicitly told to focus on an expansive ROM.

#### Static stretching protocol

Participants performed a static S&R stretch exercise (Fig. 1). The participants sat down on the ground with straight knees and lower extremities pointed forward with their feet positioned next to each other. From this position the participants leaned their trunk forward over their legs to stretch the hamstrings and lower back. Participants were allowed to pull on their shins, feet or a band connected to wall bars to increase the stretch. The participants flexed their hips until a stretch intensity of 8 on a scale of 0–10 was reached [9, 10, 41]. This stretch was held for 32 s with a rest period of 60–90 s. If the stretch intensity decreased throughout the 32 s, the participants were told to increase the stretch until the intensity was increased to 8 again. The stretch period of 32 s per set was chosen to match work time of the RT group. A period for stretching around 30 s in alignment with studies showing positive results for stretching’s effect on flexibility [6]. Like the RT group, the SS group followed a progressive structure (Fig. 1).

### Statistical analysis

All statistical analyses were conducted using IBM SPSS Statistics software (IBM Corp. Released 2017. IBM SPSS Statistics for Windows, Version 27.0. Armonk, NY: IBM Corp.). Prior to the analyses, Shapiro-Wilk tests were performed to examine the normality of the data distribution, revealing no significant deviations from normality (*p* = 0.110–0.975). To compare the groups and examine changes over time, a mixed-factorial analysis of covariance (ANCOVA) was conducted, including the pre-test results as covariates. This approach allowed for the examination of interactions between time (pre- and post-test) and group (RT, SS, CON), while controlling for baseline differences. In instances where significant differences were detected, Bonferroni post-hoc tests were employed to elucidate specific group differences. Furthermore, to assess the changes within each group from pre-test to post-test, paired samples t-tests were utilized. The significance level for all statistical tests was set at 0.05. All results are reported as mean ± standard deviation. To gauge the magnitude of effects, partial eta squared (η^2^) are reported for main and interaction effects and Hedges’ g (g) corrected effect sizes for the post-hoc tests. For η^2^, values smaller than 0.01 were considered trivial, 0.1 − 0.06 as small, 0.06–0.14 as medium and above 0.14 as a large effect while for g values smaller than 0.5 were considered trivial, 0.5–1.25 were deemed small, 1.25–1.9 were classified as moderate, and effect sizes greater than 2.0 were regarded as large [42].

## Results

### Compliance and baseline data

A mean training completion of 98.6% was reached for the RT group and 99.3% for the SS group. There were no dropouts throughout the study from any of the groups. No between-groups differences were found in any of the tests at baseline (*p* = 0.077–0.802).

### Sit and reach test

The analysis revealed a significant interaction between time and group (*p* < 0.001, η² = 0.707), indicating that the changes from pre-test to post-test differed across the intervention groups when adjusting for pre-test scores. There was also a significant main effect of time (*p* = 0.004, η² = 0.455), suggesting overall improvement in S&R scores from pre-test to post-test. Large magnitude improvements from pre- to post-test were found in RT (6.6 ± 2.6 cm, *p* = 0.002, g = 2.12) and SS (6.1 ± 1.4 cm, *p* < 0.001, g = 3.54), but not in CON (0.8 ± 1.4 cm, *p* = 0.232, g = 0.47). Post-hoc analyses using Bonferroni adjustments showed that the RT (*p* < 0.001, g = 2.53,) and SS group (*p* = 0.001, g = 2.44,) improved more than CON, while no difference was observed between the RT and SS groups (*p* = 1.000, g = 0.21,) See table 2 and figure 2 for group- and individual data.

**Table 2:** Changes from pre to post intervention in flexibility and strength tests (mean ± standard deviation)

	Control (*N* = 6)		Static stretching (*N* = 6)		Resistance training (*N* = 6)	
	Change	g	Change	g	Change	g
Sit and Reach (Cm)	0.8 ± 1.4	0.47	6.1 ± 1.4*#	3.54	6.6 ± 2.6*#	2.12
ISLDL 95% ROM (N)	49 ± 44*	0.94	−16 ± 57	0.24	145 ± 27 *#¤	4.60
ISLDL 50% ROM (N)	23 ± 27	0.73	17 ± 28	0.50	139 ± 53*#¤	2.22

*Significantly different from pre-test (*p* < 0.01), # Significant different from Control (*p* < 0.01), ¤ Significant different from Static Stretching (*p* < 0.01). Cm = centimeters, N = Newton, ISLDL = isometric straight legged deadlift, ROM = range of motion, g = effect size pre-post

### 95% ROM isometric straight legged deadlift test

A significant interaction between time and group was found for the 95% ROM isometric straight legged deadlift test (*p* < 0.001, η² = 0.695). There was no significant main effect of time (*p* = 0.341, η² = 0.065), suggesting no overall improvement in 95% ROM ISLDL MVC scores from pre-test to post-test. Despite no significant main effect of time, large magnitude improvements from pre- to post-test were found in the RT group (145 ± 27 N, *p* < 0.001, g = 4.60) with significant but small magnitude increases in the CON group (49 ± 44 N, *p* = 0.041, g = 0.94), but no change (trivial magnitude) in the SS group (-16 ± 57 N, *p* = 0.518, g = 0.24). Post-hoc analyses revealed that the RT group improved more than SS (*p* < 0.001, g = 3.36,) and CON (*p* = 0.025, g = 2.44,), while no significant difference was found between SS and CON (*p* = 0.090, g = 1.18,).

### 50% ROM isometric straight legged deadlift test

The analysis showed a significant interaction between time and group (*p* < 0.001, partial η² = 0.690), and a significant main effect of time (Wilks’ Lambda = 0.622, F [1, 14] = 8.511, *p* = 0.011, η² = 0.378). The RT group demonstrated large magnitude significant increases from pre- to post-test by 139 ± 53 N (*p* = 0.001, g = 2.22), while the small magnitude changes with SS (17 ± 28 N, *p* = 0.207, g = 0.50) and CON (23 ± 27 N, *p* = 0.088, g = 0.73) groups did not achieve significance. Post-hoc analyses revealed that the RT group achieved greater improvements than SS (*p* < 0.001, g = 2.69,) and CON (*p* = 0.003, g = 2.57,), whereas no significant difference between SS and CON were observed (*p* = 1.000, g = 0.22,).

## Discussion

The present study examined the effects of RT through an expansive ROM compared to SS effects on flexibility and maximal strength, with matched training time and stretch intensity in healthy, physically active, young adults. In accordance with the hypotheses, both the RT and SS group improved their flexibility to a similar extent, in contrast to the insignificant changes with the CON group. Importantly, the RT group improved their isometric straight legged deadlift (both 50%- and 95% ROM) significantly (large magnitude) more than the SS and CON groups.

Similar S&R flexibility improvements between the RT and SS groups might be explained by the biomechanical understanding of stretching [8]. Passive and active tension occurs while stretching or conducting RT to full ROM lengthening the connective tissue beyond resting length and torque increases [8]. Assuming that tension in extended muscle and connective tissue is needed to increase flexibility, this might explain why the RT group displayed similar improvements in S&R (hip flexion: hip extensors and lower back) flexibility compared to the SS group even though the SS groups intervention was highly specific to the S&R test. Importantly, the present study tried to match the stretch intensity and time under tension between the RT- and SS groups. Still, the SS group spent more time in the stretched position (i.e., 32 s of each set) whereas the RT had an eccentric contraction for three seconds and a concentric contraction for one second. Of note, although the tempo of the repetitions was within the recommended velocity-range for novice to intermediate practitioners [30], our findings might be a result of the prolonged eccentric contraction. It is uncertain if the same findings would have appeared with a faster repetition tempo. Furthermore, it is also unknown if the improvement in S&R flexibility was caused by structural changes to the tissue or from neural adaptations (e.g., increased stretch tolerance) [8, 10, 12, 13]. Finally, and importantly, the sample size calculation of the present study was based on detecting differences between the intervention groups and the control group. Consequently, this study may not have sufficient power to detect differences between the two intervention groups. Therefore, the findings between the intervention groups should be interpreted with caution.

The present study’s flexibility results are in contrast to Aquino et al. [25] which found no flexibility improvement from RT, and Leite et al. [23] which found no changes in flexibility from either RT or stretching. Importantly these studies conducted RT exercises with machine exercises which could limit the full ROM. For example, Aquino et al. [25] performed leg curl to full knee extension, but the torso was fixated with a hip joint at 90 degrees, which meant that the participants had no possibility of lengthening their hamstrings further if the participants was able to fully extend their knees. However, the flexibility changes from the present study do confirm the findings from Simäo et al. [19] and Morton et al. [43] which demonstrated that RT increased flexibility similar to stretching. Of note, Simäo et al. [19] included an elderly and sedentary population. Recently, a meta-analysis showed that sedentary and elderly population gain larger benefits from stretch interventions than healthy younger population [17]. To the authors knowledge, there are only two prior randomized controlled trials that have shown that healthy, physically active, young adults can improve flexibility and maximal strength from RT [20, 21]. Importantly, these studies performed RT with only the eccentric part of the movement loaded and did not have a comparative group performing a stretch intervention. Compatible with the present hypothesis, a systematic review and meta-analysis of the literature revealed similar moderate magnitude ROM increases for both RT and stretching for both sexes with no effect of age, training duration or frequency [28].

Including and matching the intensity from stretching between the SS and RT groups do not only provide novelty to this study, but may also be a key component when programming RT aiming to improve flexibility and maximal strength at the same time. The present stretch intensity scale was used in agreement with other studies measuring flexibility [9, 10]. The VAS from 0 to 10 has previously been proven to be reliable in measuring acute pain/discomfort, however, that the scale was more precise and effective at higher discomfort than at moderate discomfort [41]. This made a valid argument for choosing a relatively high (eight) discomfort measure on the VAS scale. Further when stretching and using the VAS to measure discomfort, discomfort can be interpreted as a subjective perception of stretch intensity and maximal torque tolerance [9, 10]. This made it possible to attempt to control and match the stretch intensity between groups. This might explain why the RT group in the present study found significant flexibility improvements in contrast to some other comparable studies [22, 23, 25]. To the authors knowledge, this study is the first study attempting to match training time and stretch intensity levels between the stretching- and resistance training interventions. However, it would require further studies to confirm this as a valid method, and to explore more implementations of the method.

In accordance with the hypothesis, the RT group significantly increased the straight legged deadlift maximal isometric strength more than the SS and CON group. This difference in the isometric MVC test can be explained by the RT group training resistance training for hip extensor muscles. To lower the specificity of the RT group, the intervention for a relative high repetition scheme with dynamic movements were programmed for the RT group, whereas the test was an MVC isometric pull. Even though the torque varied through the movement pattern, only the RT group improved their isometric strength significantly at both the 50% and 95% ROM from pre to post-test within groups. A previous meta-analysis demonstrated that stretching can improve strength [17] which contrast with present findings. However, the improvements were significantly larger in elderly and sedentary participants [17], and could explain why our study did not find similar results. For stretching to induce maximal strength improvements a review suggested that longer stretching time is needed, based on animal and a few human studies reviewed [44]. Of note, the CON group improved maximal strength, but only in the 95% ROM. This finding is difficult to explain, however, we cannot disregard that they had a learning effect beyond the familiarization session or that some of the participants changed their activity as a result of being allocated to the control group (i.e., a John Henry effect).

The present study has some limitations that need to be addressed. Even though the present study succeeded in increasing the time-efficiency of flexibility training in the RT group, it cannot be concluded that this is true for the maximal strength increases found in the present study, since the study did not have a traditional RT group training with less ROM to compare results. Still, dynamic resistance training through full ROM has shown to stimulate additional hypertrophy when compared to RT performed in a partial ROM [45]. Another appropriate limitation to make note is that the small number of participants in this study limits the generalization. Also, the sample size calculation was based on detecting differences between the intervention groups and the control group. Therefore, the findings between the two intervention groups should be interpreted with caution. Furthermore, the findings of the present study cannot be generalised to other populations as the participants were only included if they were healthy, young, and physically active. Further, the daily activity level outside of the intervention of the participants was not reported and was only confirmed by the participants at the beginning of the study. Although the control of daily activity level outside of the intervention was limited, the authors have no reason to speculate that daily activity levels differed much between the three groups. Furthermore, the interventions were supervised the first two weeks of the intervention with one follow-up supervision after four weeks. Consequently six weeks of the intervention were unsupervised which is not ideal. Importantly, the participants did log all their training sessions in an online document which was shared with the instructor. This helped to follow-up on each participant and make sure all participants completed the intervention within the instructions of the protocol. Notably, even though the compliance was high, since not all sessions were directly supervised, we cannot confirm whether the intervention was followed precisely as instructed.

In conclusion, this study shows that RT with an expansive ROM can improve strength more and flexibility to a similar extent as SS for young healthy and physically active adults, when training time and stretch intensity are matched.

### Practical applications

The findings of the present study suggests that when ‘time’ is a limiting factor, RT with a substantial ROM is a valid and efficient RT protocol since it increases both strength and flexibility. This should be considered by strength and exercise coaches and physiotherapist in exercise and rehabilitation situations where time is limited, and optimization of exercise is needed.

This is to the authors knowledge the first study to compare RT through an expansive ROM to SS when training time and stretch intensity between groups is matched in young, healthy and physically active adults. Future studies should focus on a (1) larger sample size and (2) on comparing the flexibility and strength effects of SS to RT with a great ROM to traditional RT, to understand how to optimize efficiency in future flexibility and strength training protocols.

## Data Availability

Data and materials can be sent on reasonable request to the corresponding author (vidar.andersen@hvl.no).

## Abbreviations

RT: Resistance training
SS: Static stretching
Con: Control
ISDL: Isometric straight legged deadlift
S&R: Sit and reach
ROM: Range of motion
ICC: Intraclass correlation coefficient
CV: Coefficient of variation
Cm: Centimetres
Kg: Kilograms
ES: Effect size
